# Nuclear magnetic resonance spectroscopy of biofluids for osteoarthritis

**DOI:** 10.1093/bmb/ldaa037

**Published:** 2020-12-08

**Authors:** Emily J Clarke, James R Anderson, Mandy J Peffers

**Affiliations:** Institute of Life Course and Medical Sciences, Musculoskeletal and Ageing Science, William Henry Duncan Building, 6 West Derby Street, Liverpool L7 8TX, UK; Institute of Life Course and Medical Sciences, Musculoskeletal and Ageing Science, William Henry Duncan Building, 6 West Derby Street, Liverpool L7 8TX, UK; Institute of Life Course and Medical Sciences, Musculoskeletal and Ageing Science, William Henry Duncan Building, 6 West Derby Street, Liverpool L7 8TX, UK

**Keywords:** nuclear magnetic resonance, osteoarthritis, biofluids, metabolomics, synovial fluid, serum, plasma, urine

## Abstract

**Background:**

Osteoarthritis is a common degenerative musculoskeletal disease of synovial joints. It is characterized by a metabolic imbalance resulting in articular cartilage degradation, reduced elastoviscosity of synovial fluid and an altered chondrocyte phenotype. This is often associated with reduced mobility, pain and poor quality of life. Subsequently, with an ageing world population, osteoarthritis is of increasing concern to public health. Nuclear magnetic resonance (NMR) spectroscopy can be applied to characterize the metabolomes of biofluids, determining changes associated with osteoarthritis pathology, identifying potential biomarkers of disease and alterations to metabolic pathways.

**Sources of data:**

A comprehensive search of PubMed and Web of Science databases using combinations of the following keywords: ‘NMR Spectroscopy’, ‘Blood’, ‘Plasma’, ‘Serum’, ‘Urine’, ‘Synovial Fluid’ and ‘Osteoarthritis’ for articles published from 2000 to 2020.

**Areas of agreement:**

The number of urine metabolomics studies using NMR spectroscopy to investigate osteoarthritis is low, whereas the use of synovial fluid is significantly higher. Several differential metabolites have previously been identified and mapped to metabolic pathways involved in osteoarthritis pathophysiology.

**Areas of controversy:**

Conclusions are sometimes conservative or overinflated, which may reflect the variation in reporting standards. NMR metabolic experimental design may require further consideration, as do the animal models used for such studies.

**Growing points:**

There are various aspects which require improvement within the field. These include stricter adherence to the Metabolomics Standards Initiative, inclusive of the standardization of metabolite identifications; increased utilization of integrating NMR metabolomics with other ‘omic’ disciplines; and increased deposition of raw experimental files into open access online repositories, allowing greater transparency and enabling additional future analyses.

**Areas timely for developing research:**

Overall, this research area could be improved by the inclusion of more heterogeneous cohorts, reflecting varying osteoarthritis phenotypes, and larger group sizes ensuring studies are not underpowered. To correlate local and systemic environments, the use of blood for diagnostic purposes, over the collection of synovial fluid, requires increased attention. This will ultimately enable biomarkers of disease to be determined that may provide an earlier diagnosis, or provide potential therapeutic targets for osteoarthritis, ultimately improving patient prognosis.

## Introduction

### Osteoarthritis

**Fig. 1:**
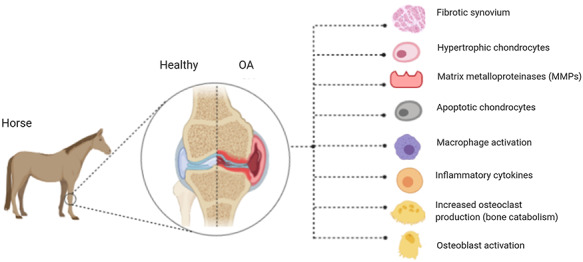
Characteristic molecular changes observed in an osteoarthritic joint. The knee joint is shown as an example.

Osteoarthritis (OA) is a common, progressive degenerative musculoskeletal pathology of synovial joints^1^ (Fig. 1). OA is inherently the result of an imbalance of catabolic and anabolic processes, often associated with disability, reduced mobility, pain and significant economic loss worldwide.^2^^,^^3^ It is of increasing concern in our ageing population, with 80% of over 65-year olds worldwide being diagnosed with the condition.^4^ OA is a complex heterogeneous condition of multiple causative factors, including mechanical, genetic, metabolic and inflammatory pathway involvement, with a non-functional joint the shared endpoint.^3^^,^^5^ In a research environment, OA is often studied using models, such as genetic, biomechanical, inflammatory and surgical,^6^ resulting in various OA phenotypes. There are cellular and molecular mechanisms of initiation and progression, resulting in loss of articular cartilage, reduced elastoviscosity of synovial fluid, thickening of subchondral bone, as well as joint space narrowing and osteophyte formation.^7^ Fundamentally, OA is a result of an imbalance in cartilage and bone remodelling. Further to this, calcified regions of cartilage are known to increase in volume and the synovium often experiences inflammation, fibrosis and vascularization, resulting in clinical symptoms such as stiffness, pain and decreased range of motion.^7^

Currently, OA is predominantly diagnosed through clinical examination and radiographic imaging, and is therefore predominantly diagnosed during later disease stages. With no disease modifying drugs currently available, management of the condition is primarily symptomatic.^3^ To manage this debilitating condition more effectively it is hoped that ongoing interrogation of OA tissues at a molecular level will identify molecules and pathways which are altered during its pathogenesis and thus identify markers for earlier diagnosis and develop novel therapeutic targets.

### Biofluids used in OA research

Blood is commonly used clinically for diagnostic purposes. It is often utilized within research as its constituents reflect systemic change within the internal environment.^8^ It is reflective of biological functionality and physiology and can therefore be used to detect impairment resulting from disease. Additionally, it is regarded as an easily obtainable sample, involving minimal stress to the patient. Serum and plasma are among the most common biofluids studied in NMR metabolomics.^9^ However, collection tubes can affect the spectra obtained, introducing additional NMR signals due to contamination.^9^ Phelan *et al.*^9^ found ethylenediaminetetraacetic acid (EDTA) tubes can result in spectral shifts and bind to various proteins within samples. Conversely, ‘Serum Z’ blood collection tubes were found to be most suited to NMR metabolomics.

Urine is produced in the kidneys through filtration, reabsorption and secretion. As a result, urine contains end products of metabolic processes occurring within the body. It has been subject to biochemical analysis for more than 100 years and is used routinely in disease diagnosis.^10^ Urine is however chemically complex which can affect analysis and downstream interpretation.^10^

The synovial membrane, which lines the inner layer of the joint capsule, consists of a one-cell thick layer of synoviocytes within a hyaluronic acid and collagen matrix and is responsible for the production of synovial fluid.^11^ The membrane provides a pool of nutrients for surrounding tissues and a medium for cellular communication with the semipermeable synovial membrane allowing passive protein transfer and synoviocytes secreting regulatory cytokines and growth factors.^12^ Due to the close relationship with articular tissues, both in terms of location and biological communication, synovial fluid provides a unique source of chemical information and holds great promise for biomarker discovery.^13^

## Metabolomics

Nuclear magnetic resonance (NMR) metabolomics has become a popular technique for the study of small molecules, known as metabolites, to understand the underlying pathophysiology of disease, enabling the identification of prospective biomarkers to inform diagnosis and therapeutic interventions. Metabolomics is the quantitative study of small molecules known as metabolites, identified within whole organisms, tissues, cells, biofluids and culture media.^1^ Metabolomics has been used to study multiple diseases associated with metabolic impairment, such as OA,^13^ alkaptonuria^14^ and cancers.^15^ The two principal analytical tools to study metabolomics are ^1^H NMR spectroscopy and mass spectrometry (MS). The aim of each study and samples utilized often dictate the most appropriate analytic method to choose. MS is sensitive, quantitative, and can provide structural information, requiring relatively low volumes and can be easily coupled to a chromatographic separation. However, ^1^H NMR spectroscopy has the additional benefits of being non-destructive, highly reproducible, with the ability to conduct targeted and untargeted analysis of both small and large molecules without any physical separation of the sample.^16^

^1^H NMR spectroscopy globally assesses the metabolome, producing individual spectra per sample that can be analysed. Sample spectra contain peaks specific to different hydrogen nuclei corresponding to various chemical groups within the small molecule metabolites. Hydrogen (^1^H) nuclei in distinct chemical groups differ from the ^1^H Larmor frequency, the result of atomic nuclei undergoing precessional motion when exposed to a magnetic field and electromagnetic radiation. Frequencies vary depending on electron shielding, resulting in a chemical shift frequency (measured in Hertz or parts per million) attributing to the varying position of peaks in the spectra.^17^ Peaks can be identified using reference databases such as the Human Metabolome Database,^18^ with the integral or area under each peak being directly proportional to the relative abundance of the metabolite within a sample.

The technique relies on a phenomenon known as NMR, which occurs when the nuclei of certain atoms, such as hydrogen (in respective metabolites) are in a static magnetic field and subjected to a short pulse of electromagnetic radiation.^19^ Atomic nuclei go from lower to higher energy states because of absorbing radiation (in the radio frequency range). This is known as selective absorption (resonance) whereby absorption only occurs if the frequency of radiation is equal to the Larmor frequency of the nuclei. The energy absorbed results in the induction of a voltage that can be detected by a coil wire, generating a current that oscillates at a given frequency.^17^ Once the electromagnetic radiation frequency pulse is removed, the oscillation will decay to zero, known as free induction decay (FID).^17^^,^^19^ A Fourier transformation (mathematical transformation) is applied to the FID signal to produce NMR spectra for analysis.^20^

## Methods

We performed a comprehensive search of PubMed and Web of Science databases using combinations of the following keywords: ‘NMR spectroscopy’, ‘Blood’, ‘Plasma’, ‘Serum’, ‘Urine’, ‘Synovial fluid’ and ‘OA’ between the years 2000 and 2020. Our focus was on experimental studies and subsequent literature; therefore reviews were excluded.

## Results

### Biofluid—blood

The use of blood has been reported in three OA studies over the last 20 years (Table 1). Experimental studies were used in all three papers, one using ovine and two murine models of OA, often surgically inducing producing a post-traumatic OA model.

**Table 1:** ^1^H NMR metabolomics studies in osteoarthritis using NMR spectroscopy, undertaken on blood samples

**Authors**	**Clinical or Experimental OA**	**Animal type**	**Study groups**	**Number of samples**	**Proton frequency**	**Total number of metabolites identified**	**Differentially abundant metabolites**	**Prospective metabolic pathways/biological context with respect to OA**
Maher *et al*, 2012^21^	Experimental	Ovine	Arthrotomy alone (sham surgery control), medial menisco-tibial ligament transection to induce meniscal destabilization, anterior cruciate ligament transaction	72, 6 per experimental group. A total of 36 samples pre surgery and 36 post surgery	800 MHz	Not mentioned	Dimethyl sulfone, 3-methylhistidine and branched chain amino acids	Anaerobic metabolism, inflammatory processes, muscle metabolism
Maerz *et al*, 2018^22^	Experimental	Murine	Control group (anaesthesia and analgesia only, no injury loading) or non-invasive ACLR group.	36, 6 per group per time point	600 MHz	58	Acylcarnitines, tryptophan glycine, carnosine, and D-mannose	Inflammatory processes and immune dysregulation to the onset and progression of PTOA following ACL injury
Mickiewicz *et al*, 2016^23^	Experimental	Murine	Wild-type and integrin α1-null mice underwent surgery to the medial meniscus. Some were then treated with the EGFR inhibitor erlotinib.	200 samples in total—100 null and 100 wild type, 50 of each sex.	600 MHz	64	O-acetylcarnitine, dimethyl sulfone, 2-hydroxyisovalerate, butyrate, trimethylamine N-oxide	Sulphur metabolism, butanoate metabolism, fatty acid metabolism, trimethylamine metabolism, valine leucine and isoleucine metabolism, inflammatory mechanisms.

*ACLR, anterior cruciate ligament rupture group; OA, osteoarthritis; PTOA, post-traumatic osteoarthritis*

Maher *et al.*^21^ analysed serum from 36 sheep to explore the metabolic phenotype of surgically induced post-traumatic OA subgroups, including a sham control, medial menisco-tibial ligament transection (MMLT) model and an anterior cruciate ligament (ACL) transection model. Dimethyl sulfone was present in increased abundance in the serum of the MMLT group. Conversely, 3-methylhistidine was more abundant, and branched chain amino acids were less abundant in the ACLT group. Differences in creatine serum levels identified were postulated to be a result of altered muscle metabolism, indicative of catabolic processes. Creatine was found in lower abundances in the MMLT group after 12 weeks, suggestive of decreased muscular metabolism. It was found following post-surgical procedures in all groups that serum levels of lactate decreased and acetate increased. Thus, observations made served to elucidate different metabolic phenotypes between models of OA, and the potential metabolic changes evident when using surgical models.

A study by Maerz *et al.*^22^ on 36 rats investigated serum metabolome differences following the induction of OA through non-invasive ACL rupture (ACLR). Using NMR spectroscopy, 58 metabolites were identified. Acylcarnitines, tryptophan, glycine, carnosine and D-mannose accounted for some of the differentially abundant metabolites between groups. Acylcarnitines were attributed to altered energy production, as it is a fatty acid ester of L-carnitine, known to promote long chain fatty acid uptake into mitochondria via the carnitine shuttle.^22^ Low levels have been associated with immune and inflammatory conditions due to incomplete beta oxidation, resulting in inflammation through the NF-kB pathway.^22^ Tryptophan metabolism was postulated to be involved as a result of the identification of decreased L-aspartic acid, 1-phenylethylamine and serotonin concentrations at 72 h, along with a decrease in L-kynurenine, serotonin, and L-aspartic acid at 4 weeks post induction. Proinflammatory pathways were hypothesized to be activated post-surgery because of reduced interleukin 17 suppression. In addition, glycine metabolism contributes to muscular fibrosis and has a regulatory purpose in collagen synthesis.^22^

In another study, Mickiewicz *et al.*^23^ analysed serum from 200 mice to quantify the effect of Erlotinib (tyrosine kinase inhibitor) treatment and the Integrin α1-Null Genotype on the serum metabolome of mice with surgically induced OA. Integrin α1 heterodimerizes with the beta 1 subunit to form a cell-surface receptor for collagen and laminin. Increased expression of integrin α1β1 and inhibition of epidermal growth factor receptor signalling have previously been shown to protect the knee from spontaneous OA. NMR spectroscopy identified 64 metabolites, with five appearing to be differentially abundant, including increased levels of O-acetylcarnitine, dimethyl sulfone, 2-hydroxyisovalerate, butyrate and trimethylamine N-oxide in the group with Erlotinib treatment and surgical OA. Dimethyl sulfone was attributed to anti-inflammatory mechanisms post-surgery. In addition, glutamine was identified as elevated in α1-Null mice compared to wild type, suggestive of protective properties, preventing reactive oxygen species induced apoptosis of chondrocytes.^23^ A decrease in serotonin was also reported, relating to tryptophan metabolism, which has been associated with OA progression in previous studies, such as in Maerz *et al.*^22^

### Biofluid—urine

Urine as a biofluid is used less frequently in OA NMR metabolomic studies with only two published research papers in the last 20 years (Table 2). Urine samples were used by Lamers *et al.*^24^ to analyse the metabolome of Hartley guinea pigs with spontaneous OA. Lactic acid, malic acid, hypoxanthine and alanine were differentially abundant and attributed to altered purine metabolism and energy production. However, limited pathway analysis and biological context were provided regarding differentially abundant metabolites within this study. This was also noted across previous studies and may reflect the change in technology and processing software available for pathway analysis. A further study by Lamer *et al.*^25^ analysed urine samples from human patients diagnosed by radiographic images. In this study, hydroxybutyrate, pyruvate, creatine and glycerol were found in greater abundance within urine from OA patients, attributed to a shift in energy production, promoting the utilization of fats. Histidine and methylhistidine were in lower abundance in OA, implicating altered histidine metabolism. Additionally, histamine and histidine decarboxylase were identified in chondrocytes derived from OA cartilage. Lamer *et al.*^25^ emphasized the lack of distinction between the local and systemic environment when using systemic fluids for metabolic research and it was suggested that this could be accounted for by experimental design, ensuring sample types are clearly distinct to the aims of the study, particularly when considering diseases, such as OA, that have a predominantly localized phenotype.

**Table 2:** ^1^H NMR metabolomics studies in OA using NMR spectroscopy, undertaken on urine samples

**Authors**	**Clinical, experimental or spontaneous OA**	**Animal type**	**Study groups**	**Number of samples**	**Proton Frequency**	**Total number of metabolites identified**	**Differentially abundant metabolites**	**Prospective metabolic pathways/biological context with respect to OA**
Lamers *et al*, 2003^24^	Spontaneous	Guinea pig	Osteoarthritic and healthy guinea pigs treated with variable vitamin C doses	44,randomly separated between different treatment groups	400 MHz	Not mentioned	Lactic acid, malic acid, hypoxanthine and alanine	Energy production and purine metabolism
Lamers *et a*l, 2005^25^	Clinical	Human	Non-OA controls and 45 individuals with radiographic OA of the knees or hips	92, 47 in non-OA group and 45 OA	600 MHz	Not mentioned	Hydroxybutyrate, pyruvate, creatine/creatinine, glycerol,histidine and methylhistidine.	Altered energy utilization, articular chondrocyte changes.

### Biofluid—synovial fluid

Synovial fluid is a popular biofluid in OA research, due to its close anatomical proximity to the diseased environment, reflecting local changes.^35^ A total of 11 papers were identified using synovial fluid to conduct NMR spectroscopy analysis (Table 3). Marshall *et al.*^32^ used an experimental canine model of OA to ascertain the effect of potential therapeutic agents on the synovial fluid metabolome. They identified increased abundance of isoleucine and decreased abundance of glucose in the specified treatment groups compared to sham controls (Table 3). They also described that arthroscopically graded OA was often accompanied by increased acetate, glucose, hydroxybutyrate and amino acids. Increased acetate is a prominent marker of polymeric degradation of cartilage and synovial fluid, whereas hydroxybutyrate was linked to the regulation of fatty acid metabolism.^32^ Interestingly, early NMR metabolomic studies did not conduct as rigorous statistical analysis and NMR spectra were compared qualitatively rather than quantitatively.^30^

**Table 3:** ^1^H NMR metabolomics studies in OA using NMR spectroscopy, undertaken on synovial fluid

**Authors**	**Clinical or experimental OA**	**Animal type**	**Study groups**	**Number of samples**	**Proton frequency**	**Total number of metabolites identified**	**Differentially abundant metabolites**	**Prospective metabolic pathways/biological context with respect to OA**
Akhbari *et al,* 2019^26^	Clinical	Human	Hip OA group and Knee OA group with ESOA	24, 12 hip and 12 knee	600 MHz	Not mentioned	N-acetylated molecules, glycosaminoglycans, citrate and glutamine	Collagen degradation, the tricarboxylic acid cycle and oxidative metabolism in diseased joints
Anderson *et al,* 2018b^27^	Clinical	Human	Knee OA and RA groups	24, 10 OA and 14 RA	700 MHz	50	32 metabolites significantly different between OA and RA SF	Glycolysis and the tricarboxylic acid cycle were lower in RA compared to OA; suggesting higher levels of inflammation, synovial proliferation and hypoxia. Elevated taurine in OA may indicate increased subchondral bone sclerosis
Anderson *et al*, 2018a^13^	Clinical	Equine	Nonseptic joints (OA and OC) and septic joint groups	18, 6 in septic group and 12 in non-septic group	600 MHz	55	Acetate, alanine, citrate, creatine phosphate, creatinine, glucose, glutamate, glutamine, glycine, phenylalanine, pyruvate, valine and glycyl proline	Extracellular matrix degradation
Hügle *et al*, 2012^28^	Clinical	Human	Gout, OA, calcium pyrophosphate disease, septic arthritis, RA, reactive arthritis, Crohn’s disease, ankylosing spondylitis, and psoriasis arthritis groups	59	600 MHz	35	Not mentioned	Not mentioned
Lacitignola *et al*, 2008^29^	Clinical	Equine	Normal/healthy and OA	33, 25 OA and 8 normal	500 MHz	Not mentioned	Acetate, alanine, acetate, N-acetylglucosamine, pyruvate, citrate, creatine/creatinine, glycerol, HDL choline, and α-glucose	Fat metabolism, cartilage and synovial fluid degradation, muscle breakdown
Mickiewicz *et al*, 2015^30^	Clinical	Human	Normal Joints (Post Mortem) and OA (Ante Mortem)	68, 55 OA and 13 control	600 MHz	55	Fructose, citrate, O-acetylcarnitine, N-phenylacetylglycine, methionine, ethanol, creatine, malate, ethanolamine, 3-hydroxybutyrate and hexanoylcarnitine	Hypoxia, inflammation and high energy requirements.
Mickiewicz *et al,* 2015^4^	Experimental	Ovine	Surgical (idealized ACL reconstruction, and sham), and non-surgical controls	18, divided into a surgical group and non-surgical group	600 MHz	65	Isobutyrate, glucose, hydroxyproline, asparagine, serine, and uridine	Chondrocyte functions and promotion of degenerative changes associated with the formation of collagen type I
Silwood and Grootveld, 2007^31^	Clinical	Human	OA group observing oxidation state and complexation status of vanadium ions in OA SF	9	700 MHz	Not mentioned	Isoleucine, leucine, valine, iso-butyrate, 3-d-hydroxybutyrate, threonine, lactate, acetate, glutamate, glutamine, pyruvate, citrate, glycine, α- and β-d-glucose, tyrosine, histidine, phenylalanine and formate.	Inflammatory processes, utilization of lipids as a source of energy
Marshall *et a*l, 2000^32^	Experimental	Canine	Hylan G-F 20 in one knee and a sham injection of saline solution in the contralateral knee (early-treatment group). The remaining six animals underwent the same treatment 2 months following the procedure (late-treatment group)	12, 6 per group	500 MHz	Not mentioned	Isoleucine	Fatty acid metabolism and polymeric degradation in cartilage and synovial fluid
Anderson *et al,* 2020^33^	Clinical	Equine	Naturally occurring OA group associated with OA grade and histological score	141	700 MHz	40	3 differentially abundant; 2-aminobutyrate, alanine and creatine	Cellular energy metabolism and collagen metabolism
Jin *et al*, 2016^34^	Clinical	Human	Three groups—traumatic diseases, infectious diseases and inflammatory diseases.	84	Not mentioned	Not mentioned	Comparison of peaks. Change in lipid peaks identified	Muscular damage/degeneration

*ESOA, end stage osteoarthritis; OC, osteochondrosis; SF, synovial fluid.*

Some large animal clinical studies have used equine synovial fluid to compare normal and OA joint metabolomes.^13^^,^^29^ In one study, lactate, alanine, glucose, acetate, pyruvate, citrate, creatine, glycerol and lipoproteins were differentially abundant, and found to be increased in OA.^29^ The group suggested increased lactate production was indicative of inflammation, while glycerol was reflective of fat metabolism. Creatinine has been implicated in muscle breakdown, which has been considered to reflect muscular breakdown in close proximity to diseased joints in OA.^29^ Acetate is a by-product of cartilage degradation and is regarded as a significant metabolite in OA pathophysiology.^29^ In a similar study, Anderson *et al.*^13^ identified a number of the same metabolites to be differentially abundant and present in higher concentrations. Glutamate, creatinine, glycine, phenylalanine, valine and glycyl proline were also identified in greater abundance in OA SF. Several of these differentially abundant metabolites related to processes such as extracellular matrix (ECM) degradation. When analysing human clinical OA samples, Mickiewicz *et al.*^30^ found 11 of the 55 metabolites identified to be differentially abundant between post-mortem and ante-mortem groups, including creatine, o-acetylcarnitine, methionine, ethanol, hydroxybutyrate and malate; all of them were found to be decreased in the OA group. It should be noted that the groups are confounding to findings from this paper, as the metabolome of deceased individuals is significantly different to that of those living. An increase in fructose and citrate in OA joints was attributed to hypoxic conditions and a potential upregulation of glucose phosphate isomerase, known to catalyse glucose 6 phosphate. Decreased malate was also linked to an altered tricarboxylic acid cycle (TCA) and the dysregulation of energy production. Furthermore, a decrease in methionine in OA groups was regarded as indicative of it being utilized and converted into s-adenosylmethionine.^30^ These have been proposed to act as cartilage damage and inflammatory reducing factors.^30^ Mickiewicz *et al.*^4^ also conducted a study on an experimental model of OA in ovine species using ACL reconstruction. In this study, metabolites such as isobutyrate, glucose, hydroxyproline and asparagine were differentially abundant. A decrease in hydroxyproline was indicative of an increase in collagen synthesis hypothesized to be in response to increased degradation.^4^ Furthermore, increased glucose abundance was related to impaired chondrocyte function, promoting degenerative changes through a disruption of dehydroascorbate transport into the cells. This resulted in an increase in reactive oxygen species and catabolic gene expression signalling pathways.^4^

Akhbari *et al.*^26^ conducted NMR analysis on human clinical samples of hip and knee OA and found citrate, n-acetylated molecules, glutamine and glycosaminoglycans to be differentially abundant in and in greater quantities in the knee group compared to hip. Citrate is an intermediate in the TCA cycle, as well as urea, amino acid and fatty acid metabolism.^26^ Altered energy production and oxidative metabolism have been regarded as characteristic of OA.^26^ Glutamine was associated with altered oxidative metabolism as well suppressing the formation of cytokines and protecting chondrocytes from heat stress. GAGs have also been regularly associated with cartilage breakdown.^26^ Anderson *et al.*^27^ observed differences between OA and rheumatoid arthritis (RA) in clinical human synovial fluid samples. They identified 32 differentially abundant metabolites between groups, including hydroxybutyrate, alanine, taurine and valine, which increased in abundance in OA. Interestingly, citrate and glutamine were also found to be increased, indicative of an altered TCA cycle and glycolysis. This was postulated to be due to the need for increased adenosine triphosphate for ECM biosynthetic machinery to function due to increased demand for ECM production in OA joints due to increased matrix metalloproteinase degradation of cartilage. In addition, taurine metabolism was implicated in OA pathophysiology, correlating with subchondral bone sclerosis, a pathogenic change related to later stage OA and its severity.

## Discussion

The number of NMR metabolomic studies of biofluids in OA has greatly increased since 2015 as the field has expanded and progressed rapidly with improved technologies and the increasing application of metabolomics. Authors of significant contribution include the Mickiewicz^4^^,^^23^^,^^30^ and Peffers^13^^,^^27^^,^^33^ groups. Many studies noted significant changes consistently in amino acids, pyruvate and creatine, with studies across all biofluids identifying a change in energy utilization, through the increased involvement of lipid metabolism, while also identifying inflammatory processes. Metabolites consistently being identified in OA metabolomics research could now be investigated in a targeted way, using mechanistic experiments to further understand their role in OA pathophysiology.

Synovial fluid has evolved to be the biofluid of preference when conducting OA research, due to its biology reflecting the local environment of the diseased joint, whereas urine and blood are systemic. The use of blood as a systemic biofluid, the collection of which is a less invasive procedure, has not yet increased sufficiently, and requires further investigation. Correlating findings between the local and systemic environment appears to be problematic due to significant confounding factors when considering the global biology of a patient, such as diet, lifestyle and co-morbidities. These variables are often controlled in studies using animal models but these models cannot fully replicate a realistic human biological environment. Additionally, the use of synovial fluid in research has been repeatedly replicated, providing scientific confidence in its use, when compared with other biofluids. Synovial fluid is favoured over urine, due to urine metabolites reflecting the global environment, rather than specific changes in the joint. Urine is also a source of end point metabolites, rather than intermediates that account for the dynamic processes occurring within the body.

Similar conclusions with respect to metabolic alteration in OA are drawn repeatedly from studies identifying similar metabolic pathways and differential metabolites, elucidating some underlying mechanisms of OA pathophysiology. However, studies are often not balanced in conclusions drawn, often not appropriately considering limitations, resulting in inflated conclusions being reported, without validation of metabolites using additional platforms or independent cohorts. The NMR metabolomic field has a limited publishing history compared to other ‘omics’ disciplines, such as proteomics, in the field of OA research. Studies are reported to varying degrees of quality, not always acknowledging relevant limitations to experimental design, such as the animal models used or the need for adequate controls. This has resulted in repeated similar studies, rather than research evolving to reflect the knowledge gained, although this is beginning to change. Anderson *et al.*^36^ recently published protocols for optimization of synovial fluid collection and processing for NMR metabolomics, providing the metabolomics community with evidence based suggestions, such as recommending synovial fluid should first be centrifuged to remove cellular material then immediately flash frozen in liquid nitrogen.^36^ As collection procedures and processing is particularly pivotal to the NMR metabolomics pipeline and can greatly affect the integrity of the sample and the metabolites identified, optimization enables increased molecular identifications and improved technical reproducibility.^36^ These factors are therefore important to consider when designing NMR metabolomics studies, including sample collection for biobanks, in order to ensure samples are suitable for NMR analysis. It also exemplifies the benefits of building on research previously conducted and identifying areas for improvement, having a significant impact on the metabolomics community, supporting the need for standardization of protocols, pipelines and ultimately quality of scientific reporting. In addition, many studies are often underpowered, with very few power calculations being recorded in the literature.

The systemic issue in the quality of metabolomics reporting has been previously addressed by the Metabolomics Society, publishing the Metabolomics Standards Initiative (MSI) in 2005.^37^^,^^38^ Here, five working groups were established to determine best practice regarding metabolomic study pipelines: biological context metadata, chemical analysis, data processing, ontology and data exchange.^35–37^ A series of papers were also published in 2007 with minimum reporting guidelines covering all areas of the metabolomics.^38^^,^^39^ Considine *et al* suggested the most significant problem in the field was data analysis, with poor adherence to guidelines by the metabolomics community and a lack of a standard for analysing metabolomics data, despite this being addressed with the development of various software such as Metaboanalyst.^39^ As a result, a lack of standardization has impaired the translational capacity, reproducibility and replicability of some metabolomic studies.

It has previously been reported within the literature that the use of silicon-based standards, such as trimethylsilyl propanoic acid (TSP) and sodium trimethylsilylpropanesulfonate (DSS), are inappropriate for use with protein-rich biofluids due to protein binding, e.g. albumins, resulting in a change in chemical shift or complete attenuation.^40^ However, almost half of the studies investigating synovial fluid within this review used TSP or DSS as an internal standard. It is also inappropriate for blood serum or plasma studies to use such reference standards for the same reasons. Despite this all NMR studies of OA using blood identified in this review used TSP or DSS. It has been postulated that providing official pipelines for preparation and analysis, as well as journals adhering to community guidelines for published work may improve the field and its current state moving forward.^39^

NMR spectroscopy of OA biofluids has provided a source of evidence implicating metabolic changes involved in the pathophysiology, initiation and progression of disease. Further studies are required to quantify and validate such findings, while adhering to metabolomics community guidelines to ensure best practice. Consideration should be given to larger study sizes, to address the issue of underpowered studies, along with more heterogeneous populations of disease to account for the variable nature of OA. This will aid in moving the field forward in the pursuit of identifying conclusive biomarkers that may be used for diagnostic purposes while exploring metabolic pathways as potential therapeutic targets. Coupling metabolomics data with other omic technologies, such as transcriptomics and proteomics, in order to take a ‘multi omic’ approach is also required to advance the field further.^41^ This will enable biological integration and interaction between different biomolecules, layering understanding and accounting for the complexity of systems biology and the dynamic environment across species. The capacity of this approach was demonstrated by Anderson *et al.*^33^ who identified a panel of molecules of interest in equine OA stratification through proteomic and metabolomic integration. It would be preferable for raw data to be provided in supplementary material sections in order to encourage transparency in research, improving communication and clarity, as over half of the cited papers in this review did not publish raw data files, or upload to data repositories. The inclusion of this would allow for additional analysis to be conducted, including meta-analysis and multivariate analysis.

## Conclusion

NMR spectroscopy of biofluids, such as serum, plasma, urine and synovial fluid, has provided further understanding of OA pathogenesis and has the capacity to enable biomarker identification. Subsequently this may allow researchers to detect early disease changes and to create specific therapeutics for OA. This can only be achieved with an increased uptake of metabolomics technologies in research, a standardized level of scientific reporting with respect to metabolomics and further exploration of the relationship between the local and global environment. We advocate the communication of best practice and robust experimental design to produce findings of significant impact that may change the scientific landscape of OA research.
